# Very-late-onset-Irvine-Gass-Syndrom

**DOI:** 10.1007/s00347-020-01266-4

**Published:** 2020-11-25

**Authors:** J. Jakob-Girbig, L. Hahner, D. Meller

**Affiliations:** Universitätsklinikum Jena, Am Klinikum 1, 07747 Jena, Deutschland

## Falldarstellung

Das Irvine-Gass-Syndrom ist eine bekannte Komplikation nach Kataraktchirurgie, die meist in relativ engem zeitlichen Zusammenhang mit der Operation auftritt. In wenigen Fällen kann dies auch noch Jahre später passieren. Aus diesem Grund sollte bei einem zystoiden Makulaödem im pseudophaken Auge auch diese Differenzialdiagnose im Rahmen einer Ausschlussdiagnostik überprüft werden.

### Anamnese

Eine 69-jährige Patientin klagte über seit 10 Tagen bestehende Sehstörungen auf dem linken Auge. Bis auf eine Kataraktoperation an diesem Auge, die 11 Jahre zuvor erfolgte, gab es bis dahin anamnestisch keine okulären Vorereignisse. Eine Lokaltherapie wurde verneint. An allgemeinen Diagnosen bestanden ein mit Apixaban antikoaguliertes Vorhofflimmern, ein Diabetes mellitus Typ II (letzter HbA_1c_ 6,2 %) sowie ein arterieller Hypertonus.

### Klinischer Befund

#### Klinische Untersuchung

**Figure 1:**
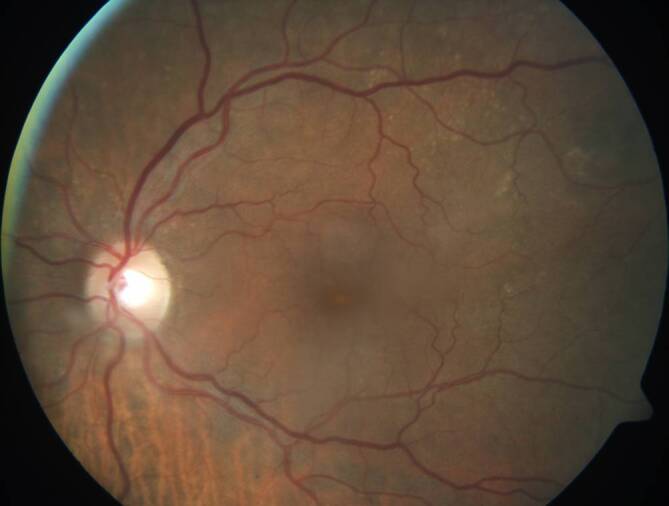

Es konnte am rechten Auge ein Visus von 0,2 LogMAR und am linken Auge von 0,6 LogMAR bei beidseitig normwertiger Tensionslage ermittelt werden. Der Vorderabschnitt gestaltete sich beidseitig reizfrei bei provekter Katarakt am rechten Auge. Am linken Auge zeigte sich eine intrakapsulär sitzende Intraokularlinse bei intakter Hinterkapsel und ohne Glaskörperprolaps. Eine Kapsulotomie war nicht durchgeführt worden. Der fundoskopische Befund rechts war unauffällig. Am linken Auge zeigten sich ein Makulaödem und wenige disseminierte harte Drusen entlang der Gefäßböden bei sonst unauffälligem Fundusbefund (Abb. 1) ohne Pigmentverschiebungen, retinale Blutungen, Exsudate, Gefäßanomalien, Glaskörpertrübungen/-zellen oder entzündliche Infiltrate der Netzhaut.

#### Optische Kohärenztomographie

**Figure 2:**
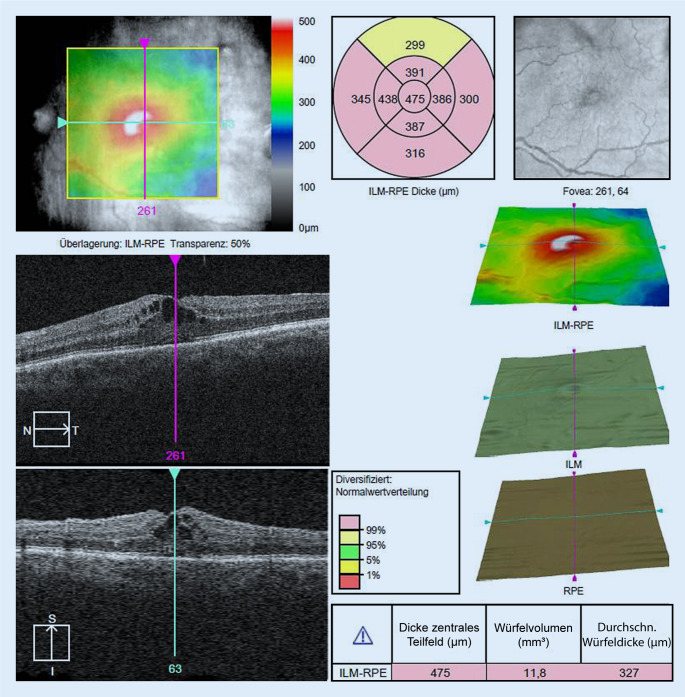

Im daraufhin durchgeführten SD-OCT der Makula ergab sich rechts ein regelhafter Makulabefund ohne Auffälligkeiten und am linken Auge der Befund eines zystoiden Makulaödems mit intraretinalen Zysten und einer deutlichen Netzhautverdickung zentral (Abb. 2).

#### Fluoreszenzangiographie

**Figure 3:**
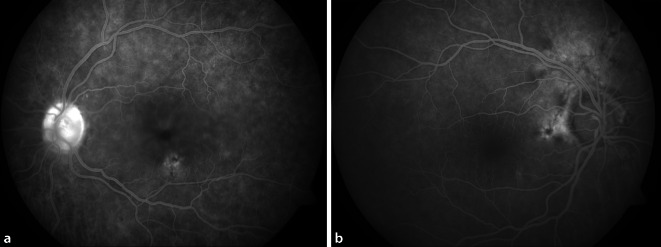

In der Fluoreszenzangiographie zeigte sich am linken Auge eine regelhafte arterielle und venöse Füllung. In der Spätphase waren eine Hyperfluoreszenz/Leckage der Papille, die Füllung zystoider Räume im Bereich der Fovea und diskrete Fensterdefekte unterhalb der Fovea zu erkennen (Abb. 3a). Am Partnerauge lagen ebenfalls Fensterdefekte (peripapillär) vor, aber keine Papillenleckage und kein Makulaödem (Abb. 3b).

### Verlauf

Fluoreszenzangiographisch ergaben sich keine Hinweise für diabetische Veränderungen, retinale Verschlussgeschehen oder choroidale Neovaskularisationen. Im OCT (und fundoskopisch) kam keine epiretinale Gliose zur Darstellung. Eine Uveitis des hinteren Augenabschnittes konnte bei fehlenden Entzündungszeichen in der Fluoreszenzangiographie und der Abwesenheit von Glaskörperzellen ausgeschlossen werden. Ohne Glaukom und fehlender topischer Medikation (Prostaglandinanaloga) kam ein Makulaödem bei Pseudophakie unter perioperativer Lokaltherapie ebenfalls nicht in Betracht. Bei vor 11 Jahren erfolgter Kataraktoperation und Vorliegen eines einseitigen zystoiden Makulaödems wurde die Diagnose eines „Very-late-onset-Irvine-Gass-Syndroms“ am linken Auge gestellt.

**Figure 4:**
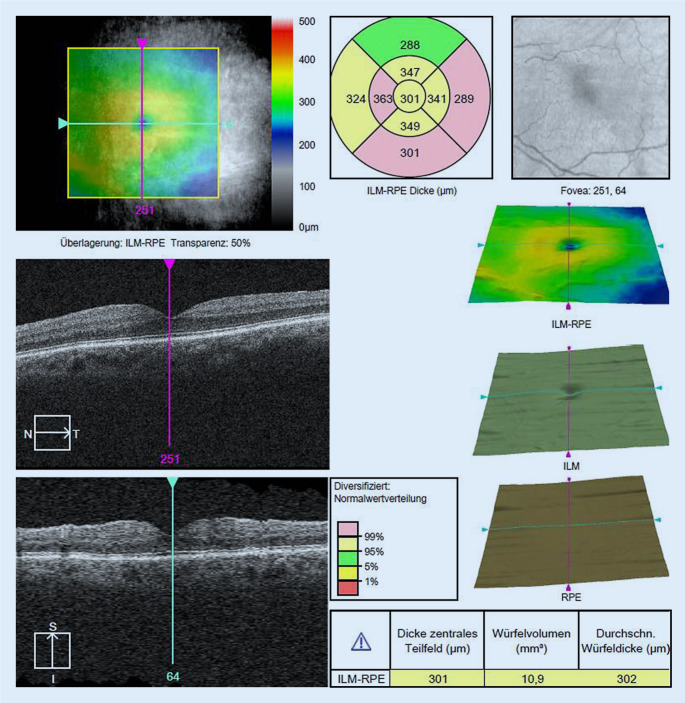

Nach der initialen Vorstellung der Patientin wurden keine therapeutischen Maßnahmen eingeleitet. Eine Verlaufskontrolle erfolgte, später als geplant, erst nach 1 Jahr. Hierbei zeigte sich ein vollständig regredienter Befund am linken Auge mit fundoskopisch und OCT-morphologisch (Abb. 4) trockener Makula mit einem Visusanstieg auf 0,2 LogMAR bei subjektiver Beschwerdefreiheit der Patientin.

## Diskussion

Das zystoide Makulaödem ist kein eigenständiges Krankheitsbild, sondern eine unspezifische Komplikation (sekundäre pathologische Entität unterschiedlicher Ätiologie). Aufgrund von Schädigungen im Bereich der Aderhaut bzw. Netzhaut kommt es zu einer intraretinalen Flüssigkeitsansammlung im Gebiet der Makula, die mit Metamorphopsien, Visusminderung, Mikropsie, Störungen des Farbsehens und Skotomen assoziiert sein kann [1].

Für die Entstehung des zystoiden Makulaödems am pseudophaken Auge scheint auf molekularer Ebene v. a. eine entzündlich bedingte Störung der Blut-Retina-Schranke ausschlaggebend zu sein [2]. Als mögliche Ursachen dessen sind Faktoren zu nennen wie Diabetes, Makuladegeneration, arterielles oder venöses Verschlussgeschehen, Lokaltherapie (v. a. Prostaglandine) oder Netzhautablösung [3].

Die Operationstechnik sowie der Operationsverlauf haben einen maßgeblichen Einfluss auf die Wahrscheinlichkeit der Entstehung eines zystoiden Makulaödems. Flach et al. beschreiben durch die intrakapsuläre Technik eine höhere Inzidenz verglichen mit der extrakapsulären Technik [4]. Bei intraoperativen Komplikationen wie Hinterkapselruptur, Glaskörperverlust, Iristrauma oder Bildung von Glaskörpersträngen am Wundspalt ist die Inzidenz laut Bradford et al. ebenfalls erhöht [5].

Der Zeitpunkt des Auftretens ist unterschiedlich. Typischerweise bildet sich ein zystoides Makulaödem 3 bis 12 Wochen postoperativ und bei 80 % der Patienten kommt es innerhalb von 3 bis 12 Monaten zur spontanen Flüssigkeitsresorption mit Visusanstieg [6]. Bei fehlender Spontanremission kommen v. a. nichtsteroidale Antiphlogistika, Steroide und Carboanhydrasehemmer zum Einsatz [8]. In der gegenwärtigen Literatur sind nur wenige Fälle des zystoiden Makulaödems beschrieben, die als „late onset“ (mehr als 3 Monate nach Operation) oder „very late onset“ (mehr als 5 Jahre nach Operation) aufgetreten sind [3, 7].

Im vorliegenden Fall konnten andere mögliche Ursachen eines zystoiden Makulaödems ausgeschlossen werden. So zeigten sich keine diabetischen oder entzündlichen Veränderungen im Bereich der übrigen Netzhaut der Patientin. Auch ein Verschlussgeschehen oder eine choroidale Neovaskularisation konnte angiographisch ausgeschlossen werden. Eine Lokaltherapie mit Prostaglandinen wurde zu keinem Zeitpunkt von der Patientin angewendet.

Aufgrund des langen Zeitraumes zwischen Operation und Auftreten des Ödems bei überschrittener gesetzlich verpflichtender Aufbewahrungsfrist konnte der Operationsbericht nicht mehr eingesehen werden, sodass diesbezüglich nur die Aussage getroffen werden kann, dass sich klinisch kein Hinweis auf eine intraoperative Komplikation bei gut sitzender Hinterkammerlinse ohne Kapseldefekt ergab.

Patienten mit stattgehabtem Irvine-Gass-Syndrom sollten bei einer Kataraktoperation am kontralateralen Auge engmaschig kontrolliert werden, da ein erhöhtes Risiko für das Auftreten eines zystoiden Makulaödems am anderen Auge besteht. Neben der strengen Indikationsstellung sollte eine komplikationslose und atraumatische Operation angestrebt werden, ein leitliniengerechtes prophylaktisches Therapieschema mit Antiphlogistika (NSAID) besteht hingegen nicht [10].

Der hier beschriebene Fall ist als einer der wenigen Very-late-onset-Fälle eines Irvine-Gass-Syndroms zu kennzeichnen und beschreibt zugleich einen Spontanverlauf mit kompletter Remission innerhalb von 12 Monaten. Auch dies stellt eine Besonderheit dar, da in den meisten Fallbeschreibungen therapeutische Interventionen erfolgt sind und bisher nur wenige Spontanverläufe dokumentiert wurden [9]. Radeck et al. vermuten weitergehend eine hohe Dunkelziffer eines günstigen Spontanverlaufs, der durch die Therapie maskiert sein könnte [10].

## Fazit für die Praxis

- Das Very-late-onset-Irvine-Gass-Syndrom ist ein seltenes Krankheitsbild mit bisher nur wenigen Fallbeschreibungen.
- Es stellt eine Ausschlussdiagnose beim zystoiden Makulaödem am pseudophaken Auge dar.
- Patienten mit Irvine-Gass-Syndrom gelten bei einer Kataraktoperation am kontralateralen Auge als Risikopatienten.
- Zur Diagnosestellung ist eine umfassende diagnostische Abklärung notwendig.
- Eine spontane Remission ist möglich.
